# Level and factors associated with uptake of human papillomavirus infection vaccine among female adolescents in Lira District, Uganda

**DOI:** 10.11604/pamj.2018.31.184.14801

**Published:** 2018-11-15

**Authors:** Esther Kisaakye, Justine Namakula, Christine Kihembo, Angela Kisakye, Peter Nsubuga, Juliet Ndimwibo Babirye

**Affiliations:** 1 School of Public Health, College of Health Sciences, Makerere University, Kampala, Uganda; 2 African Field Epidemiology Network, Lugogo House Plot 42, Lugogo Bypass, Kampala, Uganda; 3 Global Public Health Solutions, CDC EIS Program, Atlanta, Georgia

**Keywords:** Human papillomavirus vaccine, uptake, adolescents, sub-Saharan Africa, developing countries

## Abstract

**Introduction:**

the principal burden of human papillomavirus (HPV) infections is cervical cancer. Cervical cancer ranks as the fourth most common malignancy in women affecting 500,000 women each year with an estimated 266,000 deaths. Uganda has one of the highest cervical cancer incidence rates globally with an age-standardised incidence rate per 100,000 of 47.5. This study assessed the level and the factors associated with uptake of HPV vaccine by female adolescents in Lira district, Uganda.

**Methods:**

a mixed methods approach was employed using a survey among 460 female adolescents. We collected data using an interviewer-administered questionnaire. We interviewed five key informants and conducted ten in-depth interviews. Uptake was defined as completing three doses of the vaccine as per the recommended schedule. Prevalence risk ratios were used as measures of association and were computed using modified poison regression. Content analysis was used for qualitative data.

**Results:**

the mean age of the respondents was 13.97 (SD=1.24). Uptake was at 17.61% (81/460). The factors associated with uptake of HPV vaccine were: attaining ordinary level of education (aPR 1.48, 95%CI 1.11-1.97), positive attitude towards the vaccine (aPR 3.46, 95%CI 1.70-7.02), receiving vaccine doses from different vaccination sites (aPR 1.59, 95% CI 1.10-2.28) and encouragement from a health worker (aPR 1.55, 95%CI 1.15-2.11) or Village Health Team (aPR 3.47, 95%CI 1.50-8.02) to go for the vaccine. Other factors associated with uptake of HPV vaccine included; the existence of community outreaches (aPR 1.47, 95%CI 1.02-2.12), availability of vaccines at vaccination sites (aPR 4.84, 95%CI 2.90-8.08) and receiving full information about the vaccine at the vaccination site (aPR 1.90, 95%CI 1.26-2.85).

**Conclusion:**

HPV vaccine uptake was low in Lira district. Efforts to improve uptake of HPV vaccine should focus on ensuring a consistent supply of vaccines at the vaccination sites, health education aimed at creating a positive attitude towards the vaccine, sensitisation of the adolescents about the vaccine and conducting community outreaches.

## Introduction

A woman's lifetime risk of acquiring HPV infection is higher than 80%, and most infections occur within 3-4 years of sexual debut [1]. The principal burden of HPV infections globally has been cervical cancer. HPV infections are observed in over 99% of patients with cervical cancer [2]. Globally, cervical cancer is the fourth most common cancer among women affecting 500,000 women annually. In 2012, globally, there was up to 528,000 new cases of cervical cancer and 266,000 deaths [3]. In developing countries, cervical cancer accounts for 12% of female cancers and these contribute 80% of global cervical cancer cases [3]. The World Health Organization recommends HPV vaccination for girls aged 9-14 years as the most cost-effective public health intervention against cervical cancer. In Uganda, cervical cancer ranks as the most frequent cancer among women aged between 14 and 15 years. The age-standardised incidence rate of cervical cancer is high at 47.5/100,000 [4]. Despite this, many girls and women in Uganda stay without routine HPV vaccination and cervical cancer screenings because of lack of awareness, limited availability of vaccination and screening programs [5, 6]. Therefore more than three-quarters of cervical cancer patients in Uganda report with advanced disease conditions [7]. In Uganda, HPV vaccination was first piloted in 2008 in Nakasongola and Ibanda districts. It was later piloted in 12 other districts in 2012. The success of these pilot projects paved the way for countrywide scale-up of HPV vaccination before the end of 2015 and is now integrated into the national routine immunisation program [8]. Lira district is one of the first 12 districts where HPV vaccination was piloted. This study assessed the level and the factors associated with uptake of HPV vaccine by female adolescent girls in Lira district, Uganda to inform implementation of the HPV vaccine program in Uganda.

## Methods

**Study area and design:** we employed a mixed methods approach in which quantitative and qualitative data were collected using a concurrent triangulation design. By combining quantitative and qualitative data, we sought convergence and corroboration among the different data sources. The multiple perspectives provided an opportunity to develop a complete understanding of the factors associated with uptake of HPV vaccination. This study was conducted in Lira district, Northern Uganda in May 2016. Lira district is one of the 12 districts where the HPV vaccination program was first implemented (piloted) in Uganda in 2012, so the program was four years old at the time of this study.

**Study population:** the study enrolled female adolescents aged 12-17 years because they were expected to have completed the vaccination schedule.

**Quantitative study:** quantitative data were collected in a multistage cross-sectional design. The required sample size of 460 was determined using the formula by Bennett *et al.* (1991) [9] for cluster surveys with the following assumptions; a precision of 0.032 as was used in a study by Turner (2003), type I error of 5%, prevalence of uptake at 50% PATH *et al*, (2011) [10], 10 households per village and a design effect of 1.9. This study used a three-stage sampling procedure; at the first stage, we randomly selected four sub-counties out of the 13 in the district (i.e, two rural and two urban). At the second stage, we randomly selected two parishes from each of the selected sub-counties. For each parish selected, we randomly selected villages proportionate to the number of villages the parish until the required number of villages was realised. At the third stage, a list of all households with 12-17-year-old adolescent girls was generated for each village. Households from this list were randomly selected until the required sample size was realised for the village. One adolescent was selected from each household.

**Measurements:** the dependent variable was the uptake of HPV vaccine which was categorised into two; fully vaccinated and not fully vaccinated. We determined HPV uptake using respondents' recall or vaccination cards if were present. An adolescent was considered fully vaccinated if they had received all the three doses of the HPV vaccine. Any adolescent who had received one, two or no dose of HPV vaccine was considered not fully vaccinated. The Independent variables included: individual factors, community factors and health service factors. Individual factors included; sociodemographic characteristics, knowledge about the HPV vaccine and attitudes towards HPV vaccine. We measured knowledge of respondents about the HPV vaccine as a composite variable by asking respondents six questions about the HPV vaccine. The same approach has been used in other studies to measure knowledge levels [11] Table 1. The knowledge score ranged from 0-6 depending on whether the respondent had passed or failed the question. We considered scores 4-6 as good knowledge about HPV vaccination and scores 0-3 as poor knowledge [12]. We used a three-point Likert scale (2-agree, 1-neither agree nor disagree and 0-disagree) to measure attitudes of respondents towards HPV vaccination using five attitude statements as shown in Table 2. The attitude score ranged from 0-10 depending on whether the respondent agreed or disagreed to the statement. We considered scores 6-10 as positive attitude towards HPV vaccination and scores ≤ 4 as negative attitude [13].

**Table 1:** measurement of knowledge levels about the HPV vaccine of female adolescents in Lira District, Uganda

Knowledge variables	Response	Score
Had ever heard of the HPV vaccine	Yes	1
	No	0
Knew the benefits of receiving the HPV vaccine	Yes	1
	No	0
Knew the recommended doses of HPV vaccine	Yes	1
	No	0
Knew the recommended intervals between the doses of the HPV vaccine	Yes	1
	No	0
Knew the target age group for the HPV vaccine	Yes	1
	No	0
Knew where to access the vaccine from	Yes	1
	No	0

Total Score = 6, Good knowledge= scores 4-6, Poor knowledge= scores 0-3

**Table 2:** measurement of attitude levels towards the HPV vaccine among female adolescents in Lira District, Uganda

Attitude statements	Response	Score
HPV infections cause cervical cancer	Agreed	2
cervical cancer is a deadly disease	Agreed	2
it is important for young girls to receive the HPV vaccine	Agreed	2
HPV vaccine is effective in protecting girls against cervical cancer	Agreed	2
HPV vaccine has no side effects	Agreed	2

Total Score = 10, Positive attitudes = scores 6-10, Negative attitudes = scores 0-5

**Data management and analysis:** we reviewed the data for completeness, coded it, and analysed the data using STATA. We computed summary statistics like frequencies, proportions and means of different variables. We then conducted univariable, bivariable and multivariable analysis. At bivariable analysis, we computed prevalence ratios (PR) using a generalised linear model with Poisson family and a log link with robust standard errors as a measure of association because the prevalence of the outcome was above 10%. At multivariable analysis, we included all independent factors with p < 0.10 at bivariable analysis in the multivariable model to obtain adjusted prevalence risk ratios. We used the backward forward elimination with the logical model building approach of considering factors known from literature that affect HPV vaccine uptake to ascertain the best fitting model.

**Qualitative study:** for the qualitative approach, we used purposive sampling. We conducted 20 in-depth interviews, five from each category of adolescents who had not received, received one, two and three doses respectively. We also conducted five key informant interviews with the district health team members who had an expert opinion about the health service factors that influence uptake of HPV vaccination in Lira district. The district team members included the following: the assistant district health officer for maternal and child health, the district vaccines focal person, district health educator and two sub-county health facility In-charges Qualitative data were transcribed from audio recordings for content analysis. We read the transcripts several times and then coded them to identify recurring themes. We then identified key quotations that represented the central themes on factors influencing uptake of HPV vaccine and presented them in the results as text.

**Ethical considerations:** the study was approved by Makerere University School of Public Health Higher Degree Research and Ethics Committee. We sought permission to conduct the study from the Lira district and community gatekeepers. Voluntary participation in the study was sought using written informed consent.

## Results

**Social demographic characteristics of respondents:** a total of 460 respondents participated in the study with a response rate of 100%. The age of respondents ranged from 12 to 17 years with a mean age of 14 years (SD =1.24). The majority of respondents 82.6% (380/460) were living with their parents, and 94% (432/460) were in school. Most of the respondents 79.8% (367/460) had both their parents still alive (Table 3).

**Table 3:** social demographic characteristic of female adolescents aged 12–17 in Lira district, Uganda

Variable	Frequency (N=460)	Percentage (%)
**Age groups**		
12-14	306	66.5
15-17	154	33.5
**Religion**		
Catholic	186	40.4
Protestants	202	43.9
Muslims	15	3.3
Others	57	12.4
**Parent Status**		
Both alive	367	79.8
One alive	71	15.4
Both dead	22	4.8
**Living status**		
Stay with parents	380	82.6
Do not stay with parents	80	17.4
**Highest level of education**		
None	10	2.2
Primary level	252	54.8
Secondary Ordinary level	198	43.0
**Current education status**		
At school	432	93.9
Not at school	28	6.1
**Residence**		
Urban	246	53.5
Rural	214	46.5

* Mean age of 13.97 (standard deviation of 1.24)

**Level of HPV vaccine uptake:** out of the 460 respondents interviewed, 49.6%, (228/460) had not received any dose of HPV vaccine, 18.0% (83/460) had received one dose, 14.8% (68/460) had received two doses, and 17.6% (81/460) had completed all the three doses. 0ut of the 232 respondents who had initiated on the vaccine, 180 (77.6%) had received it from school. The level of uptake was 17.6% (Table 4).

**Table 4:** level of uptake, vaccination status and place of vaccination among female adolescents aged 12–17 in Lira district

Variable	Frequency (N = 460)	Percentage (%)
**Had ever visited the HPV vaccination site**		
Yes	250	54.4
No	210	45.7
**Number of doses received**		
None	228	49.6
One	83	18.0
Two	68	14.8
Three	81	17.6
**Level of uptake**		
Fully vaccinated	81	17.61
Not fully vaccinated	379	82.39
**Place of vaccination for those who had ever been vaccinated**		
School only	180	77.6
Health facility only	32	13.8
Both school and health facility	16	6.9
HPV community outreach only	04	1.7

**Knowledge about the HPV vaccination:** More than half (57.10%, 265/460) of the respondents had heard about the HPV vaccine. Half (50.87%, 234/460) of the respondents knew at least one benefit of receiving the HPV vaccine. More than half of the respondents (61.30%, 282/460) did not know the recommended doses of the HPV vaccine and the majority (81.09%, 373/460) of respondents did not know the recommended intervals between the HPV vaccine doses. More than half (53.70%, 247/460) of the respondents did not know the targeted age group for the HPV vaccine, and only 51.74% (238/460) of the respondents knew where to access the HPV vaccines. Overall, more than half of the respondents (56.09%, 258/460) had poor knowledge about the HPV vaccine (Table 5).

**Table 5:** knowledge of respondents about the HPV vaccine among female adolescents in Lira District, Uganda

Variable	Frequency	Percentage(%)
**Heard of HPV vaccine**		
Yes	265	57.10
No	195	42.39
**Knew at least one benefit of the HPV vaccine**		
Yes	234	50.87
No	226	49.13
**Knew the recommended doses of the HPV vaccine**		
Yes	178	38.70
No	282	61.30
**Knew the recommended intervals between the HPV vaccine doses**		
Yes	87	18.91
No	373	81.09
**Knew the target age group for HPV vaccines**		
Yes	213	46.30
No	247	53.70
**Knew the where to access the HPV vaccine from**		
Yes	238	51.74
No	222	48.26
**Respondent’s overall level of knowledge about HPV vaccine**		
Good	202	43.91
Poor	258	56.09

* Knowledge score ranged from 0–6, Good Knowledge = scores 4-6, Poor knowledge = scores 0-3

**Attitudes towards HPV vaccination:** the majority 70% (322/460) of the respondents agreed that HPV infections cause cervical cancer. Most of the respondents (93.7%, 431/460) agreed that cervical cancer is a deadly disease. A large proportion (88.7%, 408/460) of the respondents agreed that it is important for young adolescents to receive the HPV vaccine. Most (72.17%, 332/460) of the respondents agreed that the HPV vaccine is effective in protecting against cervical cancer. More than half (55.22%, 254/460) of the respondents agreed that the HPV vaccine has no side effects. The respondents who disagreed with the statement of HPV vaccine having no side effects gave side effects like; the injection is painful and it causes pain in the upper arms. Overall, the majority (83.48%, 384/460) of respondents had positive attitudes towards the HPV vaccine

### Factors independently associated with uptake of HPV vaccine among female adolescents aged 12-17 in Lira district

**Individual factors independently associated with uptake of HPV vaccine:** the proportion of adolescent fully vaccinated with the HPV vaccine was 48% more among adolescents who had attained a secondary ordinary level of education compared to those who had attained an education level of primary and below (Adjusted Prevalence ratio (PR) 1.48, 95%CI 1.11-1.97). Having a positive towards the HPV vaccine was a strong predictor of HPV vaccine uptake. The prevalence of uptake of HPV vaccine was 3 times higher among adolescents with positive attitudes towards the vaccine compared to those with negative attitudes (adjusted PR 3.46, 95%CI 1.70-7.02) adolescents who had received the HPV vaccine doses from more than one vaccination site had a 59% higher prevalence of HPV vaccine uptake compared to those who had received all the HPV vaccine doses from one vaccination site (adjusted PR 1.59, 95%CI 1.10-2.28). This is also revealed in a statement by one of the interviewed adolescents; *"At school, I got only two doses, but my mother took me to the hospital where I got the third dose" (In-depth interview 13).* The qualitative results from the in-depth interviews revealed that knowledge about HPV vaccination was a predictor of HPV vaccine uptake. Adolescents who had not taken any vaccination dose were not aware of the HPV vaccine and did not know the benefits. For example, most of them commonly said that; *"I am not aware of the HPV vaccine, and I don't know what it protects against"* (In-depth interview 1). However, most of the adolescents who had received one dose of the HPV vaccine were aware of the HPV vaccine but did not know the right doses and intervals between the doses. *"The teachers told us that the HPV vaccine is important and protects against cervical cancer but did not explain to us well the intervals and doses"* (In-depth interview 7) (Table 6).

**Table 6:** factors independently associated with uptake of HPV vaccine among female adolescents aged 12-17 in Lira district

Variable	HPV vaccine uptake		Un adjusted PR(95%CL)	P- value	Adjusted PR(95%CL)	P-value
	Fully vaccinated N (%)	Not fully vaccinated N (%)				
**Highest level of education**						
Secondary 0’ level	46(23.23)	152(76.77)	1.74(1.17-2.59)**	0.007	1.48(1.11-1.97)**	0.008
**Knowledge about HPV vaccine**						
Knowledgeable	55(27.23)	147(72.77)	2.70(1.76 - 4.15)***	<0.001	1.13(0.60-2.13)	0.706
**Respondent received all the vaccine doses from one site**						
Yes	11(68.75)	5(31.25)	2.12(1.45 - 3.11)***	< 0.001	1.59(1.10-2.28)*	0.013
Attitude towards HPV vaccine						
Positive	57(14.84)	327(85.16)	0.47(0.31 - 0.71)***	<0.001	3.46(1.70-7.02)**	0.001
**Respondent has ever been encouraged by a community member to go for HPV vaccine**						
Health worker						
Yes	40(37.38)	67(62.62)	3.22(2.20 – 4.70)***	< 0.001	1.55(1.15-2.11)**	0.005
VHT						
Yes	3(50.00)	3(50.00)	2.91(1.27 – 6.65)**	0.011	3.47(1.50-8.02)**	0.004
**HPV vaccine community outreaches conducted**						
Yes	16(28.07)	41(71.93)	1.74(1.09 – 2.79)*	0.021	1.47(1.02-2.12)*	0.037
**Vaccines were available at HPV vaccination site on all respondent’s visits**						
Yes	62(70.45)	26(29.55)	6.01(3.85 – 9.37)***	< 0.001	4.84(2.90-8.08)***	<0.001
**Health workers were available at HPV vaccination site on all respondent’s visits**						
						
Yes	73(35.27)	134(64.73)	1.90(0.99 – 3.64)	0.055	0.84(0.44-1.57)	0.578
**Respondent received full information about the HPV vaccine at the vaccination site**						
Yes	65(50.78)	63(49.22)	3.87(2.38 – 6.31)***	< 0.001	1.90(1.26 – 2.85)**	0.002

* Log likelihood = -120.992, Akaike’s information criterion (AIC) = 1.16, Availability of vaccines at the vaccination site on all visits, Attitude towards HPV vaccine, Highest level of education, Receiving the HPV vaccine doses from more than one site, Encouragement from health worker and VHT to go for the HPV vaccine, conducting community outreaches & Receiving full information about the HPV vaccine at the vaccination site. *Statistically significant at P<0.05

** P< 0.01

*** P< 0.001

**Community factors independently associated with uptake of HPV vaccine:** uptake of HPV vaccine was two times higher among adolescents who had been recommended or encouraged to go for the HPV vaccine by health worker compared to those who had not been recommended or encouraged by a health worker to go for the HPV vaccine (adjusted PR 1.55, 95%CI 1.15-2.11). The prevalence of uptake of HPV vaccine was three times higher among adolescents who had been encouraged by a village health team member(VHT) to go for HPV vaccination compared to those who had not been encouraged a VHT (adjusted PR 3.47, 95%CI 1.50-8.02). There was a 47% higher prevalence of uptake of HPV vaccine among adolescents who reported that HPV vaccine community outreaches were conducted in their residences compared to those who reported that the outreaches were not conducted in their residences (Adjusted PR 1.47, 95%CI 1.02-2.12).

**Health service factors independently associated with uptake of HPV vaccine:** the prevalence of uptake of the HPV vaccine was five times higher among adolescents who reported that the vaccines were available at HPV vaccination site at all their visits compared to those who reported that the vaccines were not available at all visits (Adjusted PR 4.84, 95% CI 2.90-8.08). This result is corroborated by key informants as one of them stated that; *"At first the vaccines were available but during the last lot, the vaccines became unavailable at the different vaccination sites because of inconsistent supply from the national medical store"* (Key informant interview 1). Qualitative results further revealed that there was also a challenge of lack of sufficient funds to facilitate the transport/delivery of vaccines at the different vaccination sites and the health workers responsible for vaccinating the girls *"Vaccines were sent without financial support so we had to divert some primary health care funds to facilitate those health workers who vaccinated in schools and community outreaches"* (Key informant interview 2). The prevalence of uptake of HPV vaccine was 90% higher among adolescents who received full information about the HPV vaccine at the vaccination site compared to those who did not receive full information (adjusted PR 1.90, 95%CI 1.26-2.85).

## Discussion

The study estimated the level of uptake of HPV vaccination at 18% which is below the targeted coverage of 80% required to eliminate common serotypes (i.e., 16 and 18) from targeted populations [14]. Our result is consistent with results from a study which was conducted among 10th-grade students in Berlin, Germany, 2010. In this study, HPV-vaccine uptake was low. 41.0% of respondents had received the recommended three HPV-vaccine doses [15]. Our findings reveal that attaining an education level of secondary is associated with uptake of HPV vaccine. The findings are in line with the recommendations from the World Health Organization of the vaccine targeting girls who have not started sexual debut. Most girls at secondary school level have already started sexual debut, so it is very important for them to complete the HPV vaccination before they start secondary education. Although most of the girls in Uganda do not start having sex until after they are 16.8 years of age [16], it's important that they are protected against the HPV infection in their early teenage years [17]. As indicated in the Andersen and Newman 1995 behavioural model, a positive assessment of participating in a healthy behaviour is an attraction influence towards practising that behaviour and individuals' willingness to undertake the behaviour. This model may partly explain our finding of an association between having a positive attitude towards the HPV vaccine and uptake of the vaccine in Lira district. Similar associations have been reported by a study conducted among 10th-grade students in Berlin, Germany, where findings revealed that the odds of being fully vaccinated with HPV vaccine decreased with a negative attitude toward vaccinations [15]. Interestingly, we found that receiving the HPV vaccine doses from more than one vaccination site was also a major predictor of complete HPV vaccination status. Vaccination efforts at some of the HPV vaccination settings can potentially have a markedly greater impact on overall adolescent-immunisation rates than could those at other settings [18].

A study by Ladner (2012) in the lowest income countries to assess eight HPV vaccination programs that used different modes of HPV vaccination showed that mixed models comprising school and health facility-based vaccination had better overall performance compared to models using just one of the methods [19]. In Lira district, using mixed models of HPV vaccine delivery were important in addressing missed opportunities of receiving HPV vaccination among adolescents. In our study, the prevalence of HPV vaccine uptake was higher among adolescents who had been encouraged by a health worker or a village health team member to go for HPV vaccination. This result is consistent with findings from a study by Donahue (2015) about HPV vaccine initiation among 9-13-year-olds in the United States. Findings from that study revealed that the strength of health provider recommendation regarding HPV vaccination was a particularly salient predictor of HPV vaccine initiation. Mothers were 21 times more likely to report initiation if they reported that their child´s healthcare provider discussed and recommended HPV vaccination for their child [20]. The success of HPV vaccine delivery depends on high community awareness through Information, education, communication activities as well as counselling [21]. Community health workers are noble agents of behavioural change and play a key role in the extending formal health services [22]. Therefore engaging community health workers/VHTs in community HPV mobilisation and sensitisation activities can increase acceptability of the HPV vaccine by adolescents and their parents since they are more trusted by the community members [23]. Furthermore, we found out that there was a 47% higher prevalence of HPV vaccine uptake among adolescents who reported that HPV vaccine community outreaches were conducted in their residences. This result is in agreement with results from a project by PATH (2011) in Nakasongola district, Uganda where parents reported that they were initially reluctant to have their daughters vaccinated because they did not know about cervical cancer or understand the purpose of the HPV vaccines. As they learned more from the community outreaches, they became less reluctant [8].

Findings from our study revealed that availability of HPV vaccines at vaccination sites on all adolescents' visits is associated with higher prevalence. This result is consistent with a study done by Small (2012) [24] which evaluated the impact of HPV vaccine availability on uptake among 19- to 26-year-old female patients of Planned Parenthood of Mid and South Michigan before and after the vaccine became available at the health centre. Availability of the HPV vaccine increased vaccine uptake from 11% before clinic availability to 16% after availability. Therefore, increasing the availability of the HPV vaccine at vaccination sites substantially improves HPV vaccine uptake among clients. However, improving the availability of the HPV vaccines alone is not sufficient enough for a substantial increase in uptake. Issues of accessibility, including cost and provider recommendation, must also be addressed [25]. Obtaining full information about the HPV vaccine at vaccination sites was positively associated with uptake of the vaccine. This result is consistent with results from a study by Kessels [26] to determine factors associated with HPV vaccine uptake among teenage girls. Results revealed that higher vaccine uptake was associated with having a healthcare provider as a source of information [26]. The major limitation of our study was that the assessed level of uptake might not necessarily explain the efforts of those that received only one or two doses without completing the third dose. Given the fact that we defined the uptake variable in this study as a binary outcome (i.e, fully vaccinated and not fully vaccinated). In our study, there was a possibility of poor recall in remembering the dates on which the vaccines were administered and the durations between each dose. Poor recall was minimised by cross-checking on the vaccination cards if available. The cross-sectional design that was employed limits the making of causal inferences about the main outcome and independent variables.

## Conclusion

We found out that the uptake of HPV vaccine was low. Factors found to be associated with HPV vaccine uptake included: attaining an education of secondary ordinary level, positive attitude towards the vaccine, receiving vaccine doses from more than one site and encouragement from Health workers and Village Health Teams. Other factors associated with uptake of HPV vaccine included; participating in community outreaches, availability of vaccines and receiving full information about the vaccine.

**Recommendations:** we recommended the ministry of health, Uganda to; conduct massive HPV vaccination community sensitisations to build positive attitudes, conduct HPV vaccination community outreaches and to ensure regular availability of vaccines at the vaccination sites. Further studies should also be conducted about the timeliness of HPV vaccinations among female adolescents to see the factors associated with untimely uptake of HPV vaccine doses and the implications of not receiving the doses in the correct intervals.

**Operational definitions:** human papillomavirus vaccine is the vaccine that protects against human papillomavirus which is known to prevent cervical cancer and other HPV infections in women. HPV vaccine uptake was defined as being fully vaccinated with all the three doses of HPV vaccine. Knowledge is the familiarity with information, facts, descriptions, or skills acquired through experience or education about cervical cancer and HPV vaccination. Attitude is a set of health beliefs that determine or guide individual behaviours/conduct. A household consisted of a person or group of persons, related or unrelated, who lived together in the same dwelling unit, who acknowledged one adult male or female as the head of household, who shared the same meals and living arrangements and were considered as one unit.

### What is known about this topic

- The study assessed different factors that hinder timely uptake of the HPV vaccine for instance poor attitudes towards the vaccine some of which were already known.

### What this study adds

- The study adds an in-depth understanding of the importance of timely vaccination with HPV vaccine and the implications of not receiving the vaccine in time.

## Competing interests

The authors declare no competing interests.
